# Application of Sensory Evaluation to Understand Fresh Apple Cultivar Acceptance in Kazakhstan

**DOI:** 10.3390/foods15122224

**Published:** 2026-06-20

**Authors:** Aidana Mashrapova, Bibinur Nurmanova, Zhuldyz Omarova, Alua Zeinulla, Didier Talamona, Mei Yen Chan

**Affiliations:** 1 Department of Mathematics, School of Sciences and Humanities, Nazarbayev University, Astana 010000, Kazakhstan; aidana.mashrapova@nu.edu.kz; 2 Department of Biomedical Sciences, School of Medicine, Nazarbayev University, Astana 010000, Kazakhstan; 3 Institute for Smart Systems and Artificial Intelligence (ISSAI), Nazarbayev University, Astana 010000, Kazakhstan; 4 Department of Mechanical and Aerospace Engineering, School of Engineering and Digital Sciences, Nazarbayev University, Astana 010000, Kazakhstan

**Keywords:** hedonic evaluation, triangle discrimination test, exploratory factor analysis, drivers of liking, mealiness, postharvest quality

## Abstract

Cultivar portfolio decisions and postharvest quality management in Kazakhstani fresh apple markets are made without locally validated consumer sensory benchmarks, limiting producers’ and breeders’ ability to align product design with regional consumer expectations. This exploratory study develops and pilot-tests a consumer sensory evaluation framework for fresh apple cultivars among young adults in an urban Kazakhstani context. Twenty-eight untrained adults evaluated firmness, crispness, juiciness, mealiness, sweetness, acidity, and aroma, alongside overall liking, using a 100 mm unstructured line scale, with reference-based calibration and triangle discrimination tests. Discrimination accuracy was high (96.4%; *p* < 0.001; d′ = 2.59), with no evidence of systematic anchoring bias, though this cannot be fully ruled out given the study design. Significant cultivar differences were observed for seven attributes (*p* < 0.01), with aroma showing no significant variation (*p* = 0.265). Crispness (⍴ = 0.44), sweetness (⍴ = 0.43), and juiciness (⍴ = 0.41) were the attributes most strongly and positively associated with overall liking, while mealiness exerted a negative influence (⍴ = −0.36). Exploratory factor analysis revealed three latent sensory dimensions—texture, taste, and aroma—explaining 71.22% of variance. Sex-based differences were limited to mealiness, acidity, and aroma. Given the small sample size and the absence of instrumental physicochemical measurements, these findings should be interpreted as exploratory and hypothesis-generating rather than definitive. As one of the first consumer sensory evaluation frameworks piloted in a Kazakhstani population, this study provides preliminary insights and a methodological foundation for future, larger-scale research on cultivar selection, postharvest management, and consumer-oriented product development.

## 1. Introduction

Standardised, reproducible sensory evaluation frameworks are fundamental to consumer-oriented food research, yet their development and validation for fresh unprocessed produce remains limited—particularly in populations outside Western Europe and North America [1,2]. While sensory methodologies have been extensively applied to processed foods and beverages, fresh fruit presents distinct challenges: the absence of controlled manufacturing variables, high within-cultivar variability, and the dependence of consumer perception on attributes such as texture and freshness that are difficult to standardise. These challenges are compounded in culturally underrepresented populations, where the validity of imported sensory scales cannot be assumed, and represent a critical and largely unaddressed methodological gap for Central Asian contexts.

Internationally, sensory evaluation is guided by established standards, including ISO 6658 (general sensory methodology) [3], ISO 4120 (triangle discrimination testing) [4], ISO 4121 (scaling methods) [5], ISO 13299 (sensory profiling) [6], ISO 8586 (assessor selection and training) [7], and ISO 8589 (sensory testing environments) [8]. These standards provide a widely accepted methodological foundation for consumer and analytical sensory studies. However, to the best of the authors’ knowledge, no locally validated consumer sensory evaluation framework for fresh fruit has been reported for Kazakhstan or the wider Central Asian region. This methodological gap represents a direct motivation for the present study.

A literature search of Web of Science and Scopus using the terms ‘sensory evaluation,’ ‘consumer panel,’ and ‘Central Asia’ or ‘Kazakhstan’ identified no published studies reporting a structured consumer sensory evaluation framework for this population. While isolated studies have examined food preferences and dietary patterns in the region, none have employed discrimination testing, continuous line scaling, and multivariate driver-of-liking analysis in combination with a locally recruited consumer panel. These findings suggest that the present study addresses an important gap in the regional sensory science literature.

Structured consumer sensory evaluation frameworks—combining discrimination testing, descriptive profiling, and hedonic scaling—are well established in Western European and North American populations and have been widely applied in fresh fruit research [1,9,10,11]. These include studies using descriptive sensory evaluation to characterise apple quality traits, investigations linking sensory perception with genetic and metabolomic variation among cultivars, and methodological developments in sensory characterisation techniques [9,10,11]. However, comparable structured frameworks have not been developed for Kazakhstan. Existing studies conducted in Western populations using such methodologies have consistently shown that crispness, sweetness, and juiciness are major drivers of apple acceptance, while mealy texture negatively affects consumer preference [12]. Whether similar preference patterns exist among Kazakhstani consumers remains unknown, highlighting the need for the present study.

Sensory experience is a primary determinant of consumer acceptance in fresh produce markets, where purchasing decisions depend almost entirely on the multidimensional perceptual experience of colour, texture, taste, and aroma—inputs that are cognitively and affectively processed to produce hedonic responses guiding food choice [13,14]. While instrumental measurements of firmness, soluble solids, and titratable acidity provide objective quality indicators, their correspondence with consumer perception is often limited: instrumental firmness shows only a moderate association with perceived crispness and weak relationships with perceived juiciness or sweetness [9], underscoring the need for methodologies that directly capture consumer experience. Descriptive profiling, consumer hedonic testing, and discrimination methods such as triangle tests are the principal complementary approaches in sensory science [9,10,11], yet structured and standardised methodologies remain underutilised for fresh fruit consumer assessment, restricting cross-study comparability and limiting integration of sensory data into breeding programmes, quality certification schemes, and consumer communication strategies [15].

Sensory perception is shaped not only by universal physiological mechanisms but also by cultural exposure, dietary habits, language, and genetic variation. Cross-cultural research demonstrates that taste preferences, odour perception, and hedonic responses vary significantly across populations, compromising the validity and interpretability of instruments developed elsewhere [2,14]. Genetic polymorphisms in taste receptor genes may further modulate sensitivity across ethnic groups [16]. These issues are especially acute for Central Asian populations, where the evidence base is sparse but distinctive. The distribution of bitter-taste receptor (TAS2R38) genotypes, for example, differs across the Caucasus and Central Asia, with Kazakhstan and Uzbekistan predominantly characterised by a medium-taster profile distinct from neighbouring populations [17]. At the dietary level, analysis of food preferences among over 500 individuals from six Silk Road countries—including Kazakhstan and Uzbekistan—identified culturally distinct preference clusters linked to the region’s steppe pastoralist heritage, patterns that cannot be captured by sensory instruments developed in Western contexts [18]. Without locally validated instruments, assessments risk reduced construct validity due to semantic mismatches, translation bias, or unfamiliar reference products [1]. To date, no published consumer sensory framework has been developed or validated for Central Asian populations—a gap that is particularly consequential as the region advances innovative and sustainable food product development [19,20]. The absence of locally relevant consumer sensory benchmarks represents not merely a geographical gap but also a methodological one. Sensory scales and hedonic frameworks developed in Western contexts cannot automatically be assumed to perform equivalently in populations with different dietary practices, food environments, and cultural experiences. Without local evaluation, it remains uncertain whether attribute definitions, scale anchors, and hedonic response patterns are interpreted similarly by consumers in Kazakhstan, limiting the interpretability of findings generated using imported sensory instruments.

Apples represent a particularly well-suited model system for addressing this gap. They are among the most economically significant horticultural crops in Central Asia, where fresh produce has historically been central to the daily diet and market culture [21], and they originate from a wild progenitor species native to the region [22]. Broad cultivar-level genetic diversity produces marked variation in texture and flavour attributes [9,11], making apples ideal for evaluating whether a structured protocol can reliably detect consumer-relevant perceptual differences. Sweetness and juiciness are consistently identified as strongly and positively associated with overall liking in Western populations [23], but whether these same attributes dominate preference in Central Asian consumers—or whether regional dietary history and cultivar familiarity produce a distinct hedonic structure—remains an open and important question.

The present study develops and preliminarily tests a consumer sensory evaluation framework in a Kazakhstani context, using apple cultivars as a model system, and investigates the perceptual determinants of consumer acceptance. The selected cultivars span a wide range of textural and flavour profiles representative of the Central Asian market—from the firm, acidic character of Granny Smith and Golden to the softer, sweeter profiles of Gala and Semerenko—reflecting both internationally traded varieties and cultivars with established regional presence. Semerenko and Idared have long histories of cultivation across the former Soviet republics and remain familiar to consumers in the region [21], whereas Starcrimson and Red Jonaprince represent more recently introduced commercial varieties. These cultural and market considerations provide a direct empirical basis for the hypotheses examined in the present study. Given consumers’ likely familiarity with softer, sweeter traditional cultivars alongside growing market penetration of crisper international varieties, we hypothesised that texture-related attributes—specifically crispness and firmness as measured on 100 mm continuous line scales—would emerge as attributes most strongly associated with overall liking, and that mealiness would exert a negative influence, as assessed through Spearman’s rank correlations and exploratory factor analysis. The specific aims were to: (1) evaluate the reliability and discriminative capacity of the protocol through triangle discrimination testing and reference-based calibration; (2) characterise sensory attribute profiles across cultivars and identify attributes most strongly associated with overall liking; and (3) compare the sensory attributes most strongly associated with acceptance in this Central Asian population against published Western data, identifying convergences and divergences that may reflect cultural or population-level differences in sensory preference. Beyond addressing a regional evidence gap, this study positions consumer-based sensory evaluation as a scalable methodological framework adaptable to other culturally distinct populations and fresh-food categories.

## 2. Materials and Methods

**Figure 1:**
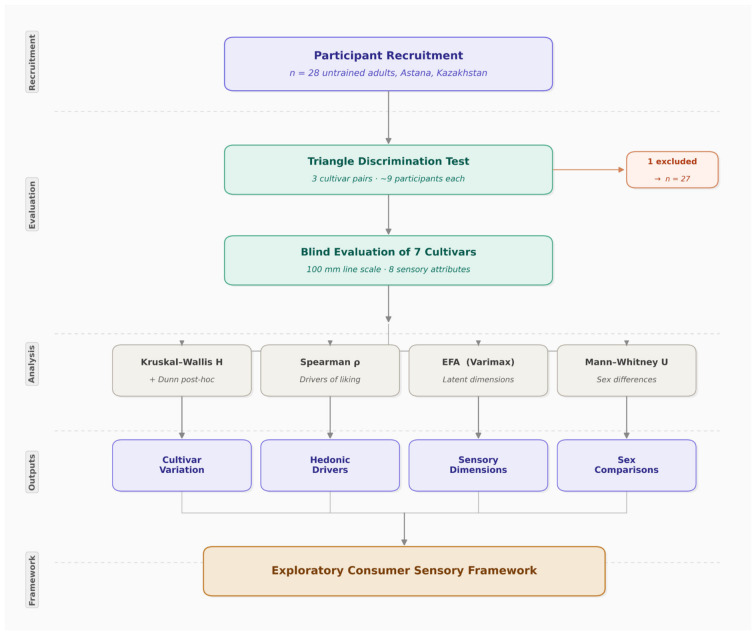
Schematic overview of the study design and analytical workflow.

An overview of the study design and analytical workflow is presented in Figure 1.

### 2.1. Participant Recruitment

Twenty-eight untrained adult participants (16 females and 12 males; age range 18–32 years, mean age 22.4 years) were recruited from the local population in Astana, Kazakhstan, using convenience sampling; no participants from neighbouring Central Asian regions or countries were included. Sample size was informed by established guidelines recommending 20–30 untrained participants for consumer discrimination panels [1,24]. Each of the 27 participants who completed the full protocol evaluated all seven apple cultivars (*n* = 27 refers to tasters throughout, not apple samples). Inclusion criteria required the absence of self-reported food allergies or oral conditions that could affect taste or texture perception. Evaluation materials, instructions, and attribute definitions were administered in three languages—Kazakh, Russian, and English—to ensure linguistic accessibility across the participant sample and to minimise the risk of semantic misinterpretation of scale anchors or attribute definitions. Participants represented a culturally homogeneous urban Kazakhstani consumer sample, consistent with this study’s objective of developing a sensory framework. All participants provided written informed consent prior to participation in accordance with institutional ethical guidelines. This study was reviewed and approved by the Nazarbayev University Institutional Research Ethics Committee under protocol number 1004/04122024.

### 2.2. Sample Preparation

**Figure 2:**
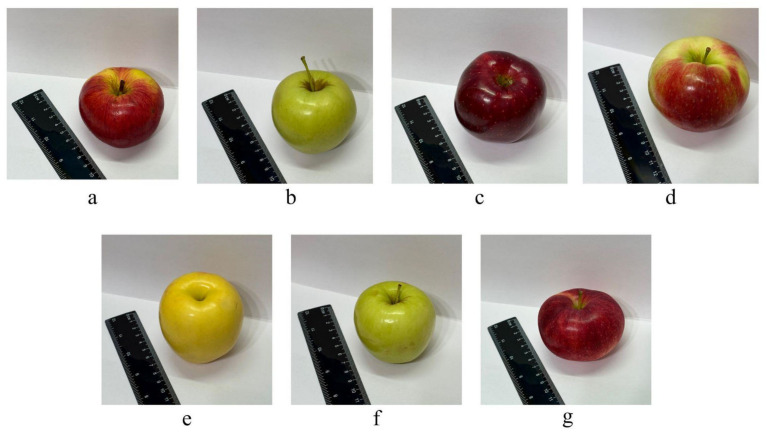
Seven apple cultivars used in this study: (**a**) Gala; (**b**) Granny Smith; (**c**) Starcrimson; (**d**) Idared; (**e**) Golden; (**f**) Semerenko; (**g**) Red Jonaprince.

Seven cultivars commonly available in the Central Asian market were selected to represent a broad range of sensory profiles: Gala, Granny Smith, Starcrimson, Idared, Golden, Semerenko, and Red Jonaprince (Figure 2). Cultivar selection was guided by regional market availability and production landscape, encompassing both internationally traded varieties and cultivars with established regional presence, ensuring that evaluated samples reflected cultivars familiar to or available to the target consumer population. All apples were sourced from the same regional commercial supplier in Astana within a one-week procurement window at the commercial ripeness stage, as defined by standard retail maturity criteria applied by the supplier. All cultivars were drawn from a single supplier order, with no multi-lot mixing. Upon receipt, samples were stored under refrigerated conditions (approximately 4 °C) and evaluated within 48 h of procurement to minimise postharvest deterioration. The samples were representative of conventionally produced fruit available through standard retail distribution channels and were not certified organic. Information on specific pre- or postharvest chemical treatments was not available from the supplier. Prior to slicing, apples within each cultivar were visually inspected and selected to be uniform in size and free of visible surface defects. Instrumental physicochemical characterisation was not performed in the present study, which represents a limitation; future work should integrate Brix and firmness measurements to complement consumer sensory data.

Prior to sensory evaluation, apples were washed, sliced into uniform semi-circular pieces (approximately 5 mm thick), and served under hygienic conditions. Uniform slice thickness was employed to standardise bite force and minimise within-sample variability. All samples were prepared using identical procedures and presented in neutral opaque containers labelled with unique randomised three-digit codes to prevent cultivar identification and minimise visual bias, as visual appearance is known to influence sensory evaluation [24]: Gala (code 279), Granny Smith (code 884), Starcrimson (code 160), Idared (code 314), Golden (code 451), Semerenko (code 753), and Red Jonaprince (code 153). Cultivar presentation order was independently randomised for each participant, such that each of the seven coded samples appeared across all ordinal positions across the panel. This minimised carryover and position effects at the sample level. Participants were instructed to cleanse their palate with water between samples.

### 2.3. Sensory Evaluation Environment and Session Procedure

Sensory evaluation sessions were conducted in a dedicated laboratory environment with individual evaluation booths, standardised lighting, and controlled temperature. Each session lasted approximately 45 min.

Participants first received standardised instructions (~5 min) covering attribute definitions, scale usage, and palate-cleansing procedures. To establish a common perceptual baseline and reduce inter-individual variation in scale usage, all participants first evaluated the Granny Smith cultivar as a reference sample (code 884) before proceeding to blind evaluation of the remaining six cultivars.

Granny Smith was selected as the reference sample because its well-characterised and perceptually distinct sensory profile—characterised by high firmness, high acidity, and relatively low sweetness—provides a consistent anchor across the range of evaluated attributes. A cultivar with clearly recognisable sensory characteristics was considered advantageous for calibrating participant use of the rating scales and promoting greater consistency in attribute interpretation among untrained consumers. We acknowledge that its pronounced acidity and firmness could, in principle, induce anchoring effects; Section 3.4 reports regression-based analyses that found no evidence of systematic reference-induced bias. Because all participants evaluated the same reference sample, these analyses should be interpreted as assessing associations consistent with potential anchoring effects rather than providing a direct test of anchoring bias.

The protocol comprised two sequential stages. First, a triangle discrimination test was conducted to assess overall consumer discriminative sensitivity across selected cultivar pairs (Starcrimson vs. Gala; Golden vs. Semerenko; Idared vs. Red Jonaprince). Participants were randomly assigned to one of the three cultivar pairs and were presented with three coded samples (two identical, one different) in a standardised order, and asked to identify the odd sample. The triangle test is a widely used sensory discrimination method that determines whether assessors can detect perceptual differences between products at a rate greater than expected by chance. In the present study, the test served as a preliminary validation of consumer discriminative ability prior to the subsequent sensory evaluation. Responses from all participants were therefore pooled, and a binomial test was used to determine whether the overall proportion of correct identifications exceeded chance expectation.

One participant who failed to correctly identify the odd sample in the triangle discrimination test was excluded from subsequent attribute-level analyses. The triangle test was used as a preliminary assessment of participants’ ability to discriminate among the apple samples before completing detailed sensory evaluations. Accordingly, successful completion of the discrimination task was considered a prerequisite for inclusion in the attribute analyses. All remaining 27 participants completed the full evaluation protocol. Although this exclusion criterion was not formally pre-specified, the rationale for exclusion is reported transparently and should be considered when interpreting the findings. Future studies should pre-register such exclusion criteria prior to data collection to avoid potential post hoc selection bias.

Second, remaining participants evaluated each of the seven cultivars using a 100 mm unstructured continuous line scale anchored at both ends (0 = lowest intensity; 100 = highest intensity) for eight sensory attributes: firmness, crispness, juiciness, mealiness, sweetness, acidity, aroma, and overall liking. Attribute definitions and scale anchors (Appendix C) were originally developed in English and translated into Russian and Kazakh by bilingual team members and reviewed by a second independent bilingual reviewer for semantic equivalence; formal back-translation was not performed. Prior to the main study, the questionnaire and sensory descriptors were pilot-tested with multilingual participants to assess clarity, comprehension, and appropriateness of the wording in Kazakh, Russian, and English. Additionally, the absence of formal back-translation means that full linguistic equivalence of descriptors across Kazakh, Russian, and English cannot be guaranteed, and future studies should consider formal back-translation procedures. During the training phase, participants were introduced to the 100 mm unstructured line scale, instructed on the interpretation of the scale anchors, and completed a practice evaluation using the reference sample before proceeding to the blinded assessments. The evaluator was available during the training phase to answer procedural and descriptor-related questions, but did not provide guidance during the formal sensory evaluation. Between each sample, participants cleansed their palates with still water and unsalted plain crackers. Attribute order was held constant within participants to reduce within-session complexity effects, though this may have introduced order-related fatigue effects for later-evaluated attributes. While cross-population differences in sensory perception, dietary practices, and food preferences have been reported in the literature, these factors were not directly measured in the present study and are discussed only as contextual motivation for evaluating consumer sensory responses in a local population.

Together, standardised attribute definitions, trilingual instructions, and reference-first calibration using Granny Smith as a common sensory anchor were designed to guide untrained participants and establish a shared evaluative baseline, consistent with established consumer panel guidelines [1,24].

### 2.4. Statistical Analysis

Line-scale responses were measured in millimetres and converted to numerical scores (0–100). Mean values and standard errors were calculated for each sensory attribute using data from the 27 participants who completed the full protocol. Descriptive statistics were used to summarise central tendency and variability.

Triangle test performance was evaluated relative to the theoretical chance level of 33.3% using a binomial test [25]. Data normality was assessed using the Shapiro–Wilk test. As the data deviated from normality, non-parametric statistical methods were applied. Cultivar differences across sensory attributes were assessed using the Kruskal–Wallis test, followed by Dunn’s post hoc tests for pairwise comparisons; *p*-values were adjusted using the Bonferroni correction to account for multiple comparisons within each attribute. Effect sizes were calculated as epsilon-squared (ε^2^) to quantify the magnitude of cultivar differences [26]. For Spearman correlation and Mann–Whitney U analyses, a conservative significance threshold of *p* < 0.05 was applied throughout to reduce the likelihood of Type I error across multiple simultaneous statistical tests. Formulas are provided in Appendix A.

Associations between sensory attributes and overall liking were examined using Spearman rank correlations, and correlation strength was interpreted according to established guidelines [27,28]. Sex-based differences in sensory ratings were examined using the Mann–Whitney U test, with sex assigned at birth (male/female) self-reported by participants.

Exploratory factor analysis (EFA) with Varimax rotation was conducted to investigate the latent sensory dimensions underlying consumer perception. Overall liking was included in the EFA to examine its structural alignment with sensory attribute dimensions rather than to treat it as a descriptive sensory variable. We acknowledge that combining hedonic and descriptive variables in a single factor solution is analytically contentious; this inclusion is justified solely on exploratory grounds, and the resulting factor loadings involving overall liking should be interpreted with particular caution. The number of factors retained was determined based on eigenvalues greater than one and the interpretability of the factor solution. Given the relatively small sample (*n* = 27), EFA results are interpreted as exploratory and hypothesis-generating rather than confirmatory. Similar factor-analytic approaches with Varimax rotation have been applied in consumer food and sensory perception research to identify latent constructs underlying responses to food-related stimuli [29,30].

Linear regression analyses were used to examine the potential influence of the reference sample on subsequent evaluations and to identify the relative contribution of individual sensory attributes to overall liking. Regression-based approaches are commonly applied in sensory and consumer research to quantify relationships between sensory attributes and consumer preference or liking [23].

All statistical analyses were performed using Python (version 3.11; Python Software Foundation, Wilmington, DE, USA) and STATA 18.0 (StataCorp LLC, College Station, TX, USA). Descriptive summaries and visualisations were generated using Microsoft Excel (Microsoft Corporation, Redmond, WA, USA).

## 3. Results

### 3.1. Triangle Test Performance

The panel demonstrated a high level of consumer discriminative sensitivity: 27 of 28 participants (96.4%) correctly identified the odd sample, a proportion significantly higher than the theoretical chance level of 33.3% (binomial test, *p* = 2.96 × 10^−13^). The chance-corrected proportion correct (Pc’ = 0.946) indicates that approximately 94.6% of correct responses were beyond what chance alone would predict. The sensitivity index d′ = 2.59—well above the threshold of 2.0 that indicates robust perceptual distinctiveness [31]—confirms that the consumer panel reliably detected perceptually meaningful differences among the evaluated cultivar pairs, supporting the exploratory discriminative capacity of the evaluation protocol.

The one participant who failed to identify the odd sample was excluded from all subsequent sensory attribute analyses according to the predefined study protocol, which required successful completion of the triangle discrimination test prior to inclusion in attribute-level analyses; all remaining 27 participants completed the full attribute evaluation and constitute the analytical sample for all subsequent results. Having confirmed the panel’s discriminative capacity, the following section characterises the sensory attribute profiles that differentiated cultivars.

### 3.2. Sensory Attribute Profiles Across Cultivars

**Figure 3:**
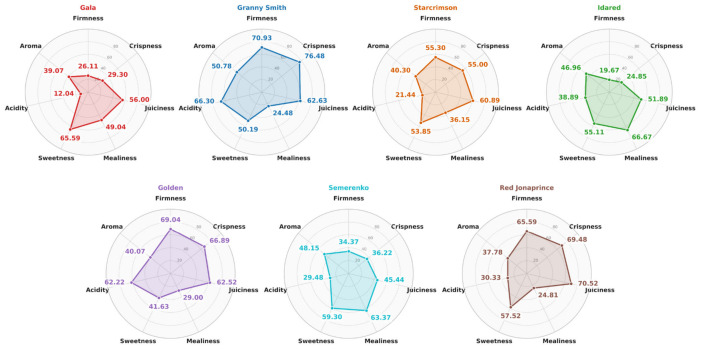
Mean sensory attribute profiles of seven apple cultivars across seven sensory attributes.

Substantial variation was observed among the seven apple cultivars across the eight sensory attributes evaluated. Table 1 reports the Kruskal–Wallis test results for each attribute, and Figure 3 presents the individual sensory attribute profiles for each cultivar. Detailed values are provided in Appendix B (Table A1).

Texture characteristics—particularly firmness and crispness—showed the largest cultivar differences by far. Granny Smith and Red Jonaprince were rated as the firmest and crispest cultivars, while Idared and Semerenko were perceived as markedly softer and more mealy. Golden sat close to Granny Smith on firmness and acidity but was notably less sweet.

At the other end of the texture spectrum, Idared and Semerenko stood out for high mealiness (mean scores 66.7 and 63.4, respectively, versus around 24–25 for the crispest cultivars), pointing to textural degradation that foreshadows the lower consumer acceptance reported in Section 3.3. Gala and Starcrimson occupied a moderate middle ground across most attributes.

Aroma was the only attribute that did not meaningfully separate the cultivars (*p* = 0.265; ε^2^ = 0.006), likely reflecting the open evaluation setting rather than true cultivar equivalence (see Section 4).

Significant cultivar differences were detected for seven of eight attributes (*p* < 0.01), with aroma being the only attribute that did not significantly discriminate among cultivars (Table 1). Texture-related attributes showed the largest effect sizes: firmness (ε^2^ = 0.434) and crispness (ε^2^ = 0.373) exhibited the strongest cultivar differentiation, followed by acidity (ε^2^ = 0.397) and mealiness (ε^2^ = 0.334), indicating that cultivar differences were most pronounced along the textural and acid taste dimensions of the sensory profile. Juiciness (ε^2^ = 0.080), overall liking (ε^2^ = 0.109), and sweetness (ε^2^ = 0.071) showed smaller but statistically significant effect sizes, suggesting moderate cultivar-level variation in these attributes.

Detailed pairwise comparisons are presented in Appendix B, Table A2.

### 3.3. Relationships Among Sensory Attributes

**Figure 4:**
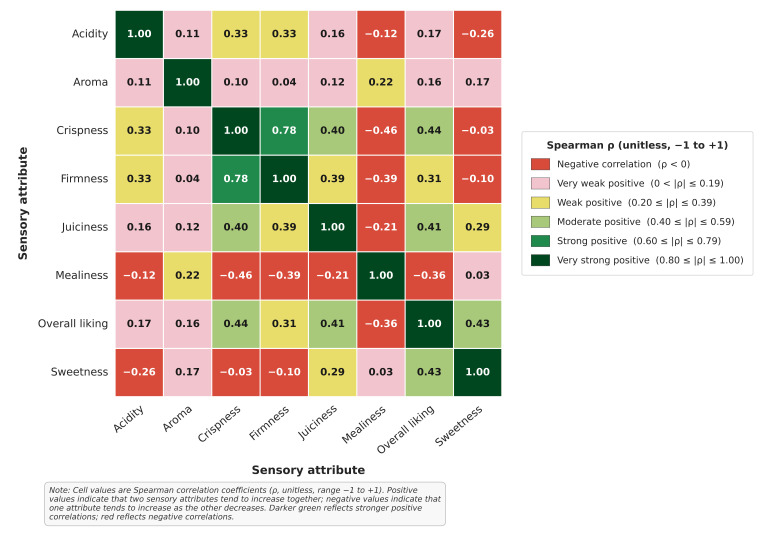
Correlation Matrix among Sensory Attributes.

The attributes consumers valued most were crispness (⍴ = 0.44, *p* < 0.01), sweetness (⍴ = 0.43, *p* < 0.01), and juiciness (⍴ = 0.41, *p* < 0.01) as the attributes most strongly and positively associated with overall liking (Figure 4). Firmness also showed a positive, albeit weaker, association (⍴ = 0.31). In contrast, mealiness exerted a consistent negative influence on overall liking (⍴ = −0.36, *p* < 0.01), representing the attribute most strongly associated with reduced consumer acceptance. Acidity (⍴ = 0.17) and aroma (⍴ = 0.16) showed very weak, non-significant associations with overall liking, indicating that at the intensities observed in this cultivar set, these attributes contributed marginally to hedonic differentiation.

These findings suggest that consumer acceptance was primarily influenced by sweetness, juiciness, and crispness, while mealiness negatively impacted perceived quality. The centrality of crispness and sweetness as positive hedonic associations is consistent with findings from consumer studies of new apple cultivars in European contexts, where insufficient crunchiness and sweetness were the attributes most strongly associated with reduced overall liking and willingness to pay [32].

Inter-attribute correlations further revealed that firmness and crispness were strongly positively associated (⍴ = 0.78), supporting their shared mechanical origin in cell-wall integrity and turgor. Both attributes were moderately correlated with juiciness (⍴ = 0.39–0.40), consistent with the mechanistic relationship between cell structure, juice release during mastication, and perceived moisture. Conversely, mealiness was negatively associated with firmness (⍴ = −0.39) and crispness (⍴ = −0.46). Sweetness showed a moderate positive association with juiciness (⍴ = 0.29), suggesting that juice release during chewing may enhance volatile-mediated sweetness perception.

### 3.4. Reference Sample Effect: Regression-Based Bias Check

To verify that Granny Smith’s distinctive profile did not distort participants’ subsequent ratings, separate linear regression models were run for each attribute, using each participant’s Granny Smith rating to predict their mean rating of the remaining six cultivars. R^2^ values ranged from 0.18 to 0.39, a level of shared variance expected within a single fruit category evaluated on identical scales. These findings suggest that the reference sample functioned primarily as a calibration tool rather than exerting a uniform influence on subsequent evaluations. However, because all participants were exposed to the same reference sample, the analyses should be interpreted as assessing associations consistent with potential anchoring effects rather than providing a direct test of anchoring bias.

### 3.5. Sex-Based Differences in Sensory Attribute Scores

**Figure 5:**
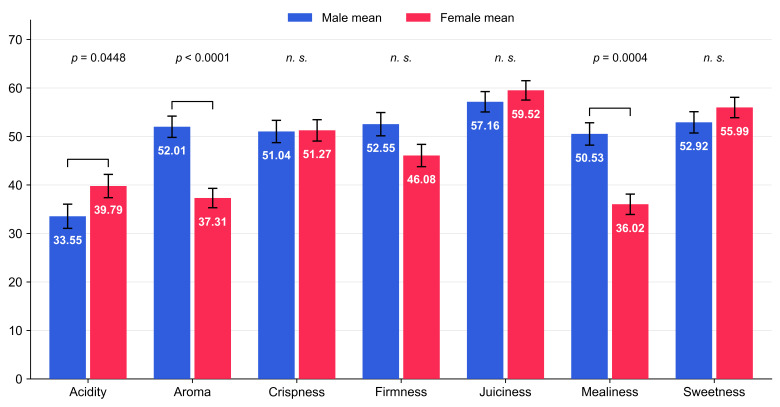
Sex-Based Differences in Apple Sensory Ratings.

Statistically significant sex-related differences were observed for mealiness (*p* = 0.0004), acidity (*p* = 0.0448), and aroma (*p* < 0.0001). Male participants rated mealiness and aroma higher than females (Figure 5). Acidity ratings were slightly higher among female participants (39.79 ± 26.61) than among male participants (33.55 ± 29.51). Critically, the attributes most strongly associated with overall liking—crispness, sweetness, and juiciness—did not differ significantly between groups, indicating that the core determinants of consumer acceptance in this population are stable across sex.

These differences should be interpreted with considerable caution due to the small sample size and potential confounding variables—including age, apple consumption habits, cultivar familiarity, and individual sensory sensitivity—which were not measured or controlled for in the present study. These findings are reported descriptively and should be regarded as exploratory observations only, requiring replication with larger, matched subgroups and appropriate covariate control before any conclusions regarding sex-based sensory differences can be drawn.

### 3.6. Exploratory Factor Analysis of Sensory Attributes of Apples

Given the exploratory nature of this study and the relatively small sample (*n* = 27), the following factor analysis results should be interpreted as preliminary and hypothesis-generating rather than confirmatory. With this caveat in mind, exploratory factor analysis identified three latent sensory dimensions explaining 71.22% of the total variance (Table 2).

Factor 1, the texture dimension, was characterised by high loadings for firmness (0.859) and crispness (0.889). Factor 2 captured taste and hedonic response, with sweetness (0.887) and overall liking (0.625) loading most strongly. Factor 3 was defined almost entirely by aroma (0.870). Detailed factor loadings are shown in Table 3.

Overall, these results suggest that consumer perception of apple sensory quality can be summarised along three factors, which together account for the majority of shared variation (71.22%) among sensory attributes.

**Figure 6:**
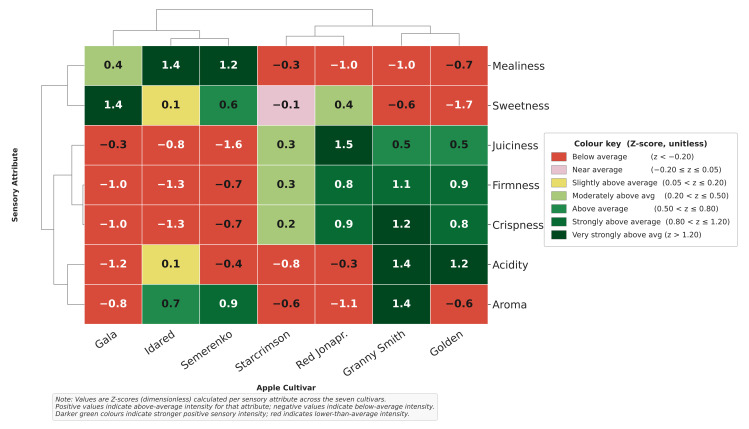
Sensory Attribute Heatmap of Apple Cultivars.

Hierarchical clustering of standardised cultivar-by-attribute scores (Figure 6) produced patterns consistent with the factor structure. Firmness and crispness clustered closely together, confirming their strong association and shared textural dimension. Juiciness showed proximity to this group, while acidity was moderately associated. In contrast, mealiness formed a distinct opposing cluster, reflecting its negative relationship with desirable texture attributes. Sweetness and overall liking formed a distinct cluster separate from the texture-related attributes, consistent with their stronger loading on the taste-hedonic factor (Factor 2) identified in the EFA rather than on the texture factor (Factor 1); this positioning suggests that hedonic evaluation in this sample was driven by taste-related perception independently of the mechanical texture dimension.

At the cultivar level, distinct clustering patterns were also observed. Granny Smith and Golden apples grouped together and were characterised by higher firmness, crispness, and acidity, indicating a texturally robust profile. Gala and Semerenko clustered separately, reflecting relatively softer textures and higher sweetness. Idared formed an isolated cluster associated with increased mealiness and lower firmness, consistent with its lower sensory preference. Red Jonaprince exhibited a distinct profile characterised by high juiciness and crispness, contributing to its higher overall liking. Starcrimson occupied an intermediate position, displaying moderate values across most sensory attributes.

Overall, the EFA and hierarchical clustering analyses tentatively suggested three sensory dimensions (texture, taste, and aroma) as a preliminary organisational pattern in consumer perception of the evaluated apple cultivars, though these should not be interpreted as confirmed or robust dimensions given the small sample size.

## 4. Discussion

The present study demonstrates that structured consumer sensory evaluation frameworks, developed and applied within this exploratory urban sample, can achieve adequate feasibility and discriminative sensitivity even with relatively small panels of untrained participants. The high discrimination accuracy observed in the triangle test (96.4%) confirms that untrained consumers are capable of detecting perceptually meaningful differences among apple cultivars, consistent with the protocol’s exploratory sensitivity to consumer-relevant variation. The absence of systematic anchoring bias associated with the reference calibration approach further supports the feasibility of this study, suggesting that perceptual standardisation can be achieved without distorting subsequent evaluations. Together, these findings suggest the framework is feasible and exploratorily applicable for consumer-oriented sensory assessment of fresh fruit in underrepresented research contexts. This is one of the first structured consumer sensory methodologies piloted for participants in this sample, and the results support its preliminary applicability in this population. Crucially, the contribution of this study extends beyond regional application: it demonstrates a structured approach for exploratorily evaluating consumer sensory methodologies in culturally distinct populations where such frameworks have not previously been established, offering a transferable model for other underrepresented food markets.

The substantive findings can be interpreted within a sensory-consumer behaviour framework, where sensory attributes act as stimuli that are integrated into a holistic perceptual experience. Rather than influencing preference independently, attributes interact through multisensory integration processes, shaping hedonic evaluation and ultimately guiding consumer acceptance. In this context, consumer perception of apple quality in this exploratory urban sample appears to be primarily structured around texture- and taste-related sensory dimensions.

Significant differences across cultivars were observed for nearly all evaluated attributes, with texture-related characteristics—particularly firmness, crispness, and mealiness—together with acidity, showing the strongest differentiation. These findings suggest that consumers rely predominantly on mechanical texture and basic taste cues when forming judgments of fruit quality. Although instrumental physicochemical characterisation was not performed in the present study, published data support the mechanistic plausibility of the observed perception patterns.

The following comparisons draw on published cultivar-level physicochemical data and are offered as a mechanistic context for the observed sensory patterns only; they do not constitute direct validation, as instrumental characterisation was not performed on the fruit batches evaluated in the present study, and published values may not reflect the specific growing conditions, harvest stage, or storage regime of the present samples. Substantial inter-cultivar variation in physicochemical parameters—including titratable acidity, soluble solids content, hardness, and volatile compound profiles—has been documented across diverse apple cultivars using chemometric approaches, confirming that sensory differences detected by consumer panels are likely underpinned by measurable compositional differences [33].

Granny Smith, which received the highest firmness and acidity ratings in the present panel, is consistent with published cultivar-level data reporting the highest titratable acidity (0.52 g malic acid per 100 g fresh weight) and high instrumental firmness (77.43 N) of commercially evaluated cultivars, alongside the lowest soluble solids content (12.2 °Brix) and pH (3.4) [34]. Golden Delicious, similarly rated highly for firmness and acidity, is consistent with reported consumer-acceptable thresholds requiring a minimum of 44 N penetrometer firmness and 3.2 g/L malate acidity to meet consumer-acceptable eating quality thresholds [35]. For Gala, consumer acceptance has been shown to depend less on firmness and acidity per se and more on juiciness and aroma [35], which may be broadly consistent with its moderate and less differentiated sensory profile in the present study, although no direct causal inference can be made. Red Jonaprince, which achieved the highest crispness and juiciness scores in our panel, is consistent with cultivar descriptions reporting firm and compact cell tissue with small cells and juicy flesh [36], though direct validation against instrumental data was not undertaken here. The elevated mealiness observed for Idared and Semerenko is consistent with published evidence linking increased water-soluble pectin galacturonic acid content during storage with sensory mealiness (R = 0.84) and reduced crunchiness and firmness [37]. At a mechanistic level, mealiness arises from loss of cell-to-cell adhesion driven by pectin solubilisation, causing tissue fracture by cell separation rather than cell rupture during mastication [37], which provides a plausible explanatory framework rather than a directly tested mechanism in this dataset. These correspondences are suggestive but cannot be taken as evidence that the consumer perceptions captured in the present study directly reflect cultivar-level physicochemical differences, as instrumental firmness, °Brix, titratable acidity, and volatile compound profiles were not measured on the same fruit batches. The observed sensory patterns are consistent with published cultivar-level data, but alternative explanations—including postharvest handling variation, storage duration differences, or lot-level variability—cannot be excluded without direct instrumental characterisation of the evaluated samples.

Consumer preference patterns were closely aligned with these sensory differences. Cultivars characterised by high firmness and crispness, such as Granny Smith and Red Jonaprince, achieved higher overall liking scores, whereas cultivars with elevated mealiness were less preferred. From a consumer behaviour perspective, crisp and juicy textures are commonly associated with freshness and quality, enhancing positive affective responses and increasing product acceptance. In contrast, mealiness appears to function as a negative sensory cue, likely due to its association with textural degradation and reduced freshness, which may trigger rejection responses. These findings are consistent with previous studies identifying mealiness as a key postharvest defect that diminishes consumer appeal [15,23], and align with recent evidence that postharvest textural degradation leading to mealiness substantially reduces consumer acceptance independently of other sensory attributes, and that interventions preserving cell wall integrity—such as 1-MCP treatment—can significantly improve overall liking by reducing mealiness while maintaining crunchiness [38].

A notable observation concerns the relative hedonic weighting of acidity. Unlike European consumer studies, where balanced sweetness-acidity ratios contribute positively to preference [23], acidity in the present sample showed a weak and non-significant association with overall liking (⍴ = 0.17), suggesting it did not meaningfully drive hedonic differentiation. Two interpretations warrant consideration. First, this may reflect a genuinely distinct hedonic weighting of sour taste among consumers in this sample, potentially linked to local dietary context and cultivar familiarity. Second, it may simply reflect the limited acidity range represented by the seven evaluated cultivars, which would suppress hedonic differentiation regardless of population-level preferences. These interpretations cannot be distinguished without studies explicitly manipulating sweetness-to-acidity ratios across a broader cultivar set or using standardised acidified stimuli.

The non-significant aroma result (*p* = 0.265) should similarly be interpreted with caution, as it may reflect methodological constraints rather than genuine cultivar-level aroma equivalence. Aroma volatiles in fresh-cut apple tissue are highly susceptible to dissipation following slicing, and the open evaluation setting—without controlled headspace, standardised exposure time, or orthonasal versus retronasal separation—may have reduced sensitivity to inter-cultivar aroma differences. Additionally, the 100 mm unstructured line scale, without aroma-specific training or reference standards, may have been insufficient to capture subtle differences that would be detectable under more controlled olfactory conditions. The non-significant aroma result should therefore be regarded as potentially reflecting a methodological artefact that cannot be distinguished from a true null effect without replication using controlled headspace sampling and trained olfactory evaluation procedures.

Sweetness, juiciness, and crispness emerged as the strongest positive contributors to overall liking, indicating that these attributes play a central role in hedonic evaluation. These sensory characteristics are closely linked to reward perception and palatability, which are critical determinants of consumer preference and food choice. Although firmness showed a positive relationship with liking, its independent contribution appeared weaker, suggesting that structural texture alone is insufficient to drive preference without complementary taste attributes. The relatively weak influence of acidity and aroma further indicates that consumers may prioritise overall sensory balance rather than the intensity of individual attributes, reinforcing the importance of integrated sensory experience.

The strong associations observed among firmness, crispness, and juiciness support the presence of cross-modal sensory interactions, whereby mechanical texture influences moisture perception and flavour release during mastication [11, 39]. At the cellular level, apple texture perception is governed by the interplay of cell turgor, cell-to-cell adhesion, and the mechanical properties of the cell wall. In firm, crisp cultivars, high turgor pressure and intact pectin-mediated cell-to-cell adhesion result in cell rupture during mastication, releasing intracellular juice and producing the acoustic and tactile sensations associated with crispness and juiciness. As postharvest storage progresses, enzymatic pectin solubilisation—particularly driven by polygalacturonase, β-galactosidase, and pectate lyase—reduces cell-to-cell adhesion, causing tissue fracture to occur preferentially by cell separation rather than cell rupture [37,40]. This shift is the primary cellular mechanism underlying mealiness: separated cells release little intracellular juice, producing the dry, grainy mouthfeel characteristic of mealy texture and simultaneously reducing perceived juiciness and crispness. The elevated mealiness observed for Idared and Semerenko is consistent with this mechanism, as both cultivars are documented to undergo pronounced pectin solubilisation during postharvest cold storage, with increases in water-soluble pectin galacturonic acid content correlating strongly with sensory mealiness (R = 0.84) and negatively with instrumental firmness [37]. These findings align with the concept that consumers integrate tactile and gustatory cues into a unified perceptual construct rather than evaluating attributes independently [9]. Such multisensory integration is a key mechanism underlying food perception and contributes directly to hedonic evaluation.

A further population-specific observation concerns the hedonic performance of cultivars with established regional familiarity. Semerenko and Idared have been among the most widely cultivated and consumed apple varieties in the former Soviet Central Asian republics for several decades and would be expected to be well-recognised by study participants. Despite this familiarity, both cultivars received comparatively lower overall liking scores, primarily driven by their elevated mealiness ratings. As noted above, elevated mealiness in these cultivars is mechanistically consistent with advanced pectin solubilisation during postharvest storage [37], suggesting that consumer dissatisfaction may reflect postharvest handling variation, storage duration differences, or cultivar-specific sensory characteristics. Because storage conditions, ripeness stage, and instrumental physicochemical parameters were not measured in the present study, these explanations cannot be disentangled. The observation that mealiness, rather than taste attributes, drove the lower acceptance of these cultivars is consistent with evidence that postharvest treatments modulating ripening progression can substantially reverse mealiness-related consumer rejection, implying that the textural deficiency is a management outcome rather than an inherent cultivar characteristic [38].

This finding tentatively suggests that cold chain management and storage optimisation may warrant investigation as potential interventions for improving the market performance of regionally important varieties, though this hypothesis requires confirmation with storage trial data. This interpretation is consistent with the ongoing dietary transition in Central Asia, where consumers appear to be increasingly receptive to textural freshness cues associated with internationally traded, crisper varieties [41], a shift that parallels trends observed in other transitioning food markets [14].

Multivariate analyses further indicated that consumer sensory perception in this exploratory urban sample can be summarised along three primary dimensions—texture, taste, and aroma—which together explained over 70% of the variance in sensory ratings. This dimensional structure suggests that consumers simplify complex sensory information into a limited number of interpretable perceptual constructs that guide evaluation and preference. The consistency between factor analysis and hierarchical clustering reinforces the robustness of these dimensions and highlights their relevance in structuring consumer perception of apple quality.

Sex-based analyses revealed largely comparable sensory evaluations between male and female participants, with statistically significant differences limited to mealiness, acidity, and aroma. While these differences may reflect variations in oral processing behaviour or perceptual sensitivity, they did not extend to the attributes most strongly associated with overall liking—crispness, sweetness, and juiciness—which did not differ significantly between groups. Descriptively, the attributes most strongly associated with overall liking did not differ between groups, though given the small sample size and absence of covariate control, no conclusions regarding sex-based differences in consumer acceptance can be drawn.

From an applied perspective, these exploratory findings suggest directions worthy of investigation for postharvest management, cultivar selection, and food product development. The strong hedonic association of crispness and juiciness raises the hypothesis that postharvest protocols aimed at preserving these attributes may be relevant for consumer acceptance, consistent with evidence that improvements in these attributes translate into measurable differences in consumer willingness to pay [12]. However, the present findings should be interpreted with caution rather than as a basis for practical recommendations, and further studies with larger and more diverse samples are needed to confirm these relationships and evaluate their implications for production and postharvest practices. The comparatively lower acceptance of familiar cultivars such as Semerenko and Idared may partly reflect postharvest handling or storage-related variation in texture quality, although cultivar-specific sensory properties may also contribute. Further studies incorporating postharvest handling and storage measurements are needed to clarify these relationships. The negligible hedonic association of acidity further suggests that breeding and import strategies targeting sweetness-acidity balance for Western markets are unlikely to translate directly to Kazakhstani consumer preferences.

Several limitations should be acknowledged. The most significant limitation of this study concerns the representativeness of the sample. The relatively small sample (*n* = 27) and the demographically narrow age range (18–32 years), drawn predominantly from a university-affiliated population, reflect an exploratory study design and limit generalisability to the broader Kazakhstani consumer population. In particular, the reliance on young, urban, and relatively well-educated participants may not capture the preferences and perceptions of older adults, rural consumers, or individuals from more diverse socioeconomic backgrounds, whose food choices and sensory expectations may differ substantially. Future studies should include larger and more diverse populations to capture potential variation across age groups, socioeconomic backgrounds, and geographic regions. In addition, while the triangle test was intended to assess overall panel discriminative sensitivity rather than discrimination between individual cultivar pairs, future studies aiming to validate pair-specific perceptual differences should recruit more participants per cultivar pair to ensure adequate statistical power for pairwise discrimination analyses. Specifically, recruitment strategies should be expanded to include older age groups, participants from rural areas, and individuals representing a wider range of income and educational levels. In addition, future studies should randomise attribute evaluation order across participants to allow for estimation and control of potential fatigue or sensory adaptation effects on later-evaluated attributes such as sweetness, acidity, and aroma.

The fixed attribute evaluation order may have introduced fatigue effects for attributes assessed later in the sequence. Additionally, the absence of instrumental physicochemical measurements, while partially addressed through reference to published cultivar data, remains a limitation. Future work should integrate instrumental physicochemical measurements—including penetrometer firmness, soluble solids content (°Brix), pH, titratable acidity, and, where feasible, volatile compound profiling—on the same fruit batches evaluated by consumers. Such data would enable direct correlation between objective quality indicators and consumer perception, clarify the mechanistic basis of the sensory differences observed here, and allow for benchmarking against published cultivar-level physicochemical profiles. Incorporation of instrumental texture analysis methods, such as texture profile analysis (TPA), would further strengthen the construct validity of consumer-reported attributes such as crispness, firmness, and mealiness.

Taken together, these results demonstrate that consumer perception of apple quality in this exploratory urban sample is driven by the integration of texture- and taste-related sensory attributes, which shape hedonic evaluation and influence acceptance. Crispness, juiciness, and sweetness emerged as the attributes most strongly associated with overall liking, while mealiness negatively affected perceived quality. In broad terms, this pattern is consistent with findings from Western sensory research [11,23], suggesting that the core architecture of apple sensory preference may be relatively universal. However, the present study also identifies meaningful local specificities: the negligible hedonic association of acidity, the low liking of texturally degraded traditional cultivars despite their regional familiarity, and the absence of meaningful aroma differentiation across cultivars collectively suggest that the relative weighting of sensory attributes is modulated by local dietary context, product availability, and potentially population-level sensory sensitivity differences. These nuances would not have been detectable without a locally validated sensory instrument, underscoring the value of regionally specific sensory frameworks. Despite its limitations, this study provides a preliminary framework requiring replication that can be extended in future regional research, and offers an exploratory cross-cultural sensory baseline against which future Central Asian food studies can be anchored.

## 5. Conclusions

This study demonstrates that consumer perception of apple quality in this exploratory urban sample is shaped by the integrated effects of texture- and taste-related sensory attributes, which together form a coherent sensory experience that underpins hedonic evaluation. The high discrimination accuracy achieved by untrained consumers, combined with robust and consistent sensory profiles across cultivars, supports the feasibility of consumer-based sensory methodologies for fresh fruit quality assessment in this exploratory sample. Critically, comparison of the present findings with published sensory data from Western consumer populations reveals both universal and population-specific features. The strong positive hedonic associations of crispness, juiciness, and sweetness are broadly consistent with Western literature. However, the weak hedonic influence of acidity, the low liking of texturally degraded traditional cultivars despite their regional familiarity, and the absence of meaningful aroma differentiation suggest that consumers in this sample weigh sensory attributes in ways that are not fully predicted by cross-cultural extrapolation from Western studies, though these patterns require replication with larger and more diverse populations before any regional conclusions can be drawn. These findings underscore the necessity of locally validated sensory instruments and baseline data for underrepresented populations, particularly as the Central Asian food market undergoes a rapid dietary transition.

Crispness, juiciness, and sweetness emerged as attributes most strongly associated with overall liking, indicating that attributes associated with freshness and palatability play a central role in shaping positive hedonic responses. In contrast, mealiness was associated with reduced acceptance, suggesting that negative textural cues can diminish the overall sensory experience. Acidity showed only a weak and non-significant association with overall liking, indicating that it did not meaningfully contribute to hedonic differentiation within the range of cultivars evaluated. Exploratory factor analysis tentatively suggested that consumer perception may be organised along three latent dimensions—texture, taste, and aroma—though given the small sample size (*n* = 27), this structure should be regarded as hypothesis-generating only and requires replication with larger, more representative samples before any confirmatory interpretation can be made.

From a food design perspective, these exploratory findings suggest several directions worthy of investigation in future research. The strong hedonic association of crispness and juiciness generates the hypothesis that postharvest protocols aimed at preserving these attributes may influence consumer acceptance, although this possibility requires confirmation through larger-scale consumer studies combined with instrumental quality measurements. The comparatively lower acceptance of familiar cultivars such as Semerenko and Idared, driven primarily by elevated mealiness, raises the hypothesis that differences in postharvest handling and storage conditions may contribute to consumer perceptions of quality; however, this interpretation cannot be evaluated without controlled storage experiments. Similarly, the weak hedonic association of acidity in this sample suggests that the relative importance of acidity may differ across populations, generating a testable hypothesis for future cross-cultural sensory research. More broadly, the proposed methodology offers a potential framework for linking sensory perception with hedonic response in this Central Asian population.

Beyond fresh apples, the consumer sensory evaluation framework presented here may be transferable to other fresh fruit categories, such as stone fruits, citrus, and berries, where cultivar-level sensory variation is similarly high, and consumer benchmarks are limited. The same protocol, comprising reference-based calibration, triangle discrimination testing, continuous line scaling, and multivariate analysis, could be applied with appropriate adaptation to animal products such as fresh or processed meat, dairy, and fish, where texture- and taste-related attributes are likewise central to consumer acceptance. More broadly, the three-stage analytical approach employed here—exploratory study, attribute profiling, and driver-of-liking analysis—provides a replicable template for consumer sensory studies seeking to establish locally relevant benchmarks in culturally distinct populations. Researchers working in underrepresented food markets could apply this framework to generate region-specific sensory baselines, support cultivar or product selection decisions, and contribute to a more globally representative evidence base in consumer food science.

## Figures and Tables

**Table 1:** Cultivar differences across sensory attributes (Kruskal–Wallis test, *n* = 27).

Sensory Attribute	χ^2^ (df = 6)	*p*-Value	Effect Size (ε^2^)
Firmness	90.872	0.0001	0.434
Crispness	79.073	0.0001	0.373
Juiciness	20.085	0.0012	0.080
Mealiness	69.356	0.0001	0.334
Sweetness	18.450	0.0052	0.071
Acidity	82.254	0.0001	0.397
Aroma	7.648	0.2650	0.006
Overall liking	24.994	0.0003	0.109

**Table 2:** Variance Explained by Extracted Factors from Exploratory Factor Analysis of Apple Sensory Attributes.

Factor	Eigenvalue	% of Total Variance	Cumulative %
1	2.8446	35.56	35.56
2	1.6332	20.41	55.97
3	1.2200	15.25	71.22

**Table 3:** Rotated Factor Loadings (Varimax Rotation) of Apple Sensory Attributes.

Variable	Factor 1	Factor 2	Factor 3
Firmness	**0.859**	−0.057	−0.004
Crispness	**0.889**	0.062	−0.005
Juiciness	0.547	0.486	0.157
Mealiness	−0.612	−0.108	0.563
Sweetness	−0.110	**0.887**	0.105
Acidity	0.577	−0.420	0.331
Aroma	0.072	0.155	**0.870**
Overall liking	0.542	**0.625**	0.032

Note: Bold values indicate dominant loadings (|loading| > 0.60).

## Data Availability

The datasets generated during and/or analysed during the current study are available from the corresponding author on reasonable request.

## Appendix A

The Kruskal–Wallis test statistic is defined as follows:

(A1) $$H=\frac{12}{N(N+1)}\sum \frac{R_{j}^{2}}{n_{j}}-3(N+1)$$

where N is the total number of observations, $n_{j}$ is the number of observations in group j, and $R_{j}$ is the sum of ranks in a group j.

Effect size epsilon-squared is calculated as follows:

(A2) $$\varepsilon^{2}=\frac{H-k+1}{N-k}$$

where H is the Kruskal–Wallis test statistic, k is the number of groups, and N is the total sample size.

Spearman’s rank correlation is defined as follows:

(A3) $$⍴=1-\frac{6\sum d_{i}^{2}}{n(n^{2}-1)}$$

where $d_{i}$ is the difference between the ranks of corresponding values and n is the number of observations.

The Mann–Whitney U statistic is calculated as follows:

(A4) $$U=n_{1}n_{2}+\frac{n_{1}(n_{1}+1)}{2}-R_{1}$$

where $n_{1}$ and $n_{2}$ are the sample sizes of the two groups, and $R_{1}$ is the sum of ranks for group 1.

The sensitivity index $d^{\prime }$ used to evaluate the triangle test performance is defined as follows:

(A5) $$d^{\prime }=z(H)-z(FA)$$

where z(H) is the z-score corresponding to the hit rate and z(FA) is the z-score corresponding to the false alarm rate.

## Appendix B

**Table A1:** Mean (±SE) sensory scores of seven apple cultivars across seven sensory attributes.

Attribute	Granny Smith	Gala	Starcrimson	Idared	Golden	Semerenko	Red Jonaprince
Firmness	70.93 ± 3.96	26.11 ± 2.88	55.30 ± 5.47	19.67 ± 3.18	69.04 ± 3.99	34.37 ± 3.88	65.59 ± 4.29
Crispness	76.48 ± 3.47	29.30 ± 4.23	55.00 ± 5.31	24.85 ± 4.49	66.89 ± 3.95	36.22 ± 4.73	69.48 ± 3.96
Juiciness	62.63 ± 3.72	56.00 ± 3.92	60.89 ± 3.70	51.89 ± 5.28	62.52 ± 4.74	45.44 ± 4.31	70.52 ± 3.06
Mealiness	24.48 ± 2.83	49.04 ± 4.44	36.15 ± 4.78	66.67 ± 4.23	29.00 ± 3.45	63.37 ± 4.90	24.81 ± 3.47
Sweetness	50.19 ± 4.01	65.59 ± 3.69	53.85 ± 4.26	55.11 ± 4.51	41.63 ± 4.12	59.30 ± 4.58	57.52 ± 4.02
Acidity	66.30 ± 4.39	12.04 ± 2.39	21.44 ± 3.60	38.89 ± 4.24	62.22 ± 4.80	29.48 ± 3.91	30.33 ± 4.46
Aroma	50.78 ± 4.27	39.07 ± 4.33	40.30 ± 4.33	46.96 ± 4.48	40.07 ± 4.28	48.15 ± 4.73	37.78 ± 4.82

**Table A2:** Dunn’s post hoc pairwise comparisons of sensory attribute scores among apple cultivars. Only statistically significant comparisons (*p* < 0.05) are shown.

Criterion	Apple Types	z-Test	*p*-Value
Firmness	153 vs. 279	4.940741	<0.0001
Firmness	160 vs. 279	3.656024	0.0001
Firmness	153 vs. 314	5.840417	<0.0001
Firmness	160 vs. 314	4.555700	<0.0001
Firmness	160 vs. 451	−1.795614	0.0363
Firmness	279 vs. 451	−5.451638	<0.0001
Firmness	314 vs. 451	−6.351314	<0.0001
Firmness	153 vs. 753	3.935148	<0.0001
Firmness	160 vs. 753	2.650431	0.0040
Firmness	314 vs. 753	−1.905270	0.0284
Firmness	451 vs. 753	4.446044	<0.0001
Firmness	160 vs. 884	−2.033617	0.0210
Firmness	279 vs. 884	−5.689641	<0.0001
Firmness	314 vs. 884	−6.589317	<0.0001
Firmness	753 vs. 884	−4.684047	<0.0001
Crispness	153 vs. 160	1.839140	0.0329
Crispness	153 vs. 279	4.848302	<0.0001
Crispness	160 vs. 279	3.009162	0.0013
Crispness	153 vs. 314	5.531126	<0.0001
Crispness	160 vs. 314	3.691986	0.0001
Crispness	279 vs. 451	−4.467017	<0.0001
Crispness	314 vs. 451	−5.149841	<0.0001
Crispness	153 vs. 753	4.048351	<0.0001
Crispness	160 vs. 753	2.209211	0.0136
Crispness	451 vs. 753	3.667066	0.0001
Crispness	160 vs. 884	−2.761202	0.0029
Crispness	279 vs. 884	−5.770364	<0.0001
Crispness	314 vs. 884	−6.453188	<0.0001
Crispness	753 vs. 884	−4.970413	<0.0001
Juiciness	153 vs. 160	1.650027	0.0495
Juiciness	153 vs. 279	2.511833	0.0060
Juiciness	153 vs. 314	2.814899	0.0024
Juiciness	153 vs. 753	4.038388	<0.0001
Juiciness	160 vs. 753	2.388361	0.0085
Juiciness	451 vs. 753	2.742562	0.0030
Juiciness	753 vs. 884	−2.634057	0.0042
Mealiness	153 vs. 279	−3.535425	0.0002
Mealiness	160 vs. 279	−1.963295	0.0248
Mealiness	153 vs. 314	−5.655684	<0.0001
Mealiness	160 vs. 314	−4.083553	<0.0001
Mealiness	279 vs. 314	−2.120259	0.0170
Mealiness	279 vs. 451	2.821613	0.0024
Mealiness	314 vs. 451	4.941872	<0.0001
Mealiness	153 vs. 753	−5.184792	<0.0001
Mealiness	160 vs. 753	−3.612661	0.0002
Mealiness	279 vs. 753	−1.649367	0.0495
Mealiness	451 vs. 753	−4.470980	<0.0001
Mealiness	279 vs. 884	3.532934	0.0002
Mealiness	314 vs. 884	5.653192	<0.0001
Mealiness	753 vs. 884	5.182301	<0.0001
Sweetness	160 vs. 279	−2.006242	0.0224
Sweetness	279 vs. 314	1.781802	0.0374
Sweetness	153 vs. 451	2.553626	0.0053
Sweetness	160 vs. 451	1.983798	0.0236
Sweetness	279 vs. 451	3.990040	<0.0001
Sweetness	314 vs. 451	2.208238	0.0136
Sweetness	451 vs. 753	−2.896520	0.0019
Sweetness	279 vs. 884	2.522453	0.0058
Acidity	153 vs. 279	2.754303	0.0029
Acidity	160 vs. 279	1.475609	0.0700
Acidity	279 vs. 314	−3.971929	<0.0001
Acidity	160 vs. 314	−2.496321	0.0063
Acidity	153 vs. 451	−3.932048	<0.0001
Acidity	160 vs. 451	−5.210743	<0.0001
Acidity	279 vs. 451	−6.686351	<0.0001
Acidity	314 vs. 451	−2.714422	0.0033
Acidity	279 vs. 753	−2.807894	0.0025
Acidity	451 vs. 753	3.878458	0.0001
Acidity	153 vs. 884	−4.404392	<0.0001
Acidity	160 vs. 884	−5.683087	<0.0001
Acidity	279 vs. 884	−7.158695	<0.0001
Acidity	314 vs. 884	−3.186766	0.0007
Acidity	753 vs. 884	−4.350802	<0.0001
Aroma	153 vs. 884	−2.088898	0.0184
Aroma	279 vs. 884	−1.839477	0.0329
Aroma	451 vs. 884	−1.707284	0.0439
Overall	153 vs. 160	1.768925	0.0385
Overall	153 vs. 314	2.812329	0.0025
Overall	153 vs. 753	2.258839	0.0119
Overall	160 vs. 884	−3.216227	0.0006
Overall	279 vs. 884	−2.656504	0.0039
Overall	314 vs. 884	−4.259631	<0.0001
Overall	451 vs. 884	−2.821055	0.0024
Overall	753 vs. 884	−3.706141	0.0001

## Appendix C

### Appendix C.1. Apple Tasting Instructions

Participants were presented with seven apple samples labelled with three-digit codes. Apple 884 was used as a reference sample and was evaluated first to establish a perceptual baseline. All other samples were evaluated relative to the reference. Palate cleansing with water was performed between samples.

### Appendix C.2. Sensory Evaluation

Participants evaluated each apple sample using a 100 mm continuous line scale anchored at 0 and 100.

**Table A3:** Consumer sensory evaluation questionnaire (verbatim excerpt).

Sensory Attribute	Definition	100 mm Continuous Line Scale
Firmness	Firmness refers to the perceived hardness of the apple when bitten, indicating how much resistance it provides.	0—Very soft 100—Very firm
Crispness	Crispness refers to the breaking sensation when biting into the apple, often accompanied by a distinct crunchy sound.	0—Not crispy 100—Very crispy
Juiciness	Juiciness measures the amount of liquid released when chewing and reflects water content and freshness.	0—Very dry 100—Very juicy
Mealiness	Mealiness describes the grainy, dry, or powdery texture of the apple flesh.	0—Not mealy 100—Very mealy
Sweetness	Sweetness refers to the perceived sugar level of the apple.	0—Not sweet 100—Very sweet
Acidity	Acidity measures the sharpness or tartness of the apple caused by organic acids.	0—Not acidic 100—Very acidic
Aroma	Aroma refers to the intensity of the apple’s smell, including fruity, floral, or herbal notes.	0—No aroma 100—Very aromatic
Overall impression	Overall impression reflects the general preference for the apple considering all sensory characteristics.	0—Did not like it 100—Liked it very much

### Appendix C.3. Triangle Test

Participants were presented with three coded apple samples (two identical and one different) and asked:

• Which apple is different from the other two? _______
• Which two apples are the same? ______
